# Multi-Hops Functional Connectivity Improves Individual Prediction of Fusiform Face Activation via a Graph Neural Network

**DOI:** 10.3389/fnins.2020.596109

**Published:** 2021-01-14

**Authors:** Dongya Wu, Xin Li, Jun Feng

**Affiliations:** ^1^School of Information Science and Technology, Northwest University, Xi’an, China; ^2^School of Mathematics, Northwest University, Xi’an, China; ^3^State-Province Joint Engineering and Research Center of Advanced Networking and Intelligent Information Services, School of Information Science and Technology, Northwest University, Xi’an, China

**Keywords:** multi-hops connectivity, graph neural network, individual prediction, connectivity–function relationship, fusiform face function

## Abstract

Brain connectivity plays an important role in determining the brain region’s function. Previous researchers proposed that the brain region’s function is characterized by that region’s input and output connectivity profiles. Following this proposal, numerous studies have investigated the relationship between connectivity and function. However, this proposal only utilizes direct connectivity profiles and thus is deficient in explaining individual differences in the brain region’s function. To overcome this problem, we proposed that a brain region’s function is characterized by that region’s multi-hops connectivity profile. To test this proposal, we used multi-hops functional connectivity to predict the individual face activation of the right fusiform face area (rFFA) via a multi-layer graph neural network and showed that the prediction performance is essentially improved. Results also indicated that the two-layer graph neural network is the best in characterizing rFFA’s face activation and revealed a hierarchical network for the face processing of rFFA.

## Introduction

Brain connectivity acts as the pathway for transferring information between brain regions and determines the information inflow and outflow of each cortical region. Passingham et al. (2002) proposed that the function of each cortical region can be determined by the region’s input and output connectivity profiles. Mars et al. (2018) further tested and extended this proposal via the neuroimaging of connectivity, and showed that the connectivity space composed by each region’s connectivity profiles provides a powerful framework in describing a brain region’s function.

The connectivity profile can be defined in terms of the white matter pathway represented by tractography through diffusion magnetic resonance imaging (MRI), or in terms of the temporal coupling between spontaneous fluctuations of resting-state functional MRI (rfMRI) signal. Under the proposal of Passingham et al. (2002), previous studies have utilized structural connectivity (Johansen-Berg et al., 2004; Tomassini et al., 2007; Beckmann et al., 2009; Saygin et al., 2011a) or functional connectivity (Cohen et al., 2008; Gordon et al., 2016) to characterize the boundary of functionally distinct brain regions, or have utilized structural connectivity (Saygin et al., 2011b; Osher et al., 2016; Saygin et al., 2016; Wu et al., 2020) or functional connectivity (Tavor et al., 2016; Parker Jones et al., 2017) to predict the functional activation information of brain regions at various task states. Though the proposal that a brain region’s function is represented by the input and output connectivity profiles is widely adopted in various studies, this proposal is deficient in characterizing the individual differences of a target brain region’s function. Specifically, under this proposal, a brain region’s function can be represented by a linear combination of the region’s connectivity profiles. This representation only utilizes direct connectivity profiles and neglects the individual differences in the functional information of neighboring regions. Since these individual differences in neighboring regions can also transfer to the target brain region via the direct connections, neglecting these individual differences is not beneficial for characterizing target brain region’s function.

**FIGURE 1:**
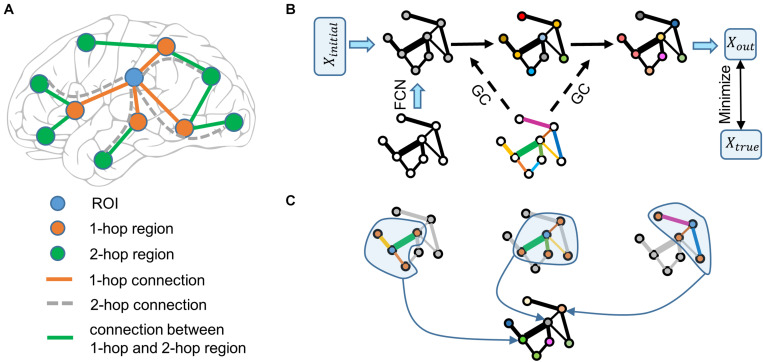
Schematic illustration of the graph neural network. **(A)** Functional information of the ROI (blue node) is transferred from the 1-hop regions (orange nodes) via the 1-hop connections (orange edges), and the functional information of the 1-hop regions is transferred from the 2-hop regions (green nodes) via 1-hop regions’ connections (green edges). The 2-hop regions indirectly connect to the ROI via 1-hop regions through the 2-hop connections (only four examples of gray dashed edges are shown). The dashed line indicates that the connection does not really exist. **(B)** Node color represents the functional information of brain regions. Edge width represents the strength of functional connectivity pathways. Edge color represents the coefficient of graph convolution (GC), i.e., the extent to which each connection participates in the functional information propagation. Initial functional information (a vector of ones) transfers within the functional connectivity network (FCN) through the graph convolution network. Coefficients of GC are trained by minimizing the error between the predicted output and true functional activation. **(C)** Three examples of graph convolution computation are shown. Central nodes (blue) integrate functional information from 1-hop regions (orange) to update its information. Graph convolution coefficients (edge color) indicate the extent to which each connection (edge width) participates in the functional information propagation.

To overcome this problem, we proposed that more connectivity features in the brain connectivity network should be considered. Explicitly, as shown in Figure 1A, functional information of the region of interest (ROI) is transferred from 1-hop regions (direct neighbors of the ROI) and the information of 1-hop regions is unknown, however, the information of 1-hop regions is transferred from 2-hop regions (direct neighbors of 1-hop regions) through the connections of 1-hop regions. Therefore, even though 2-hop regions do not connect to the ROI directly, they can affect the ROI via 1-hop regions. Denote the direct connections between the ROI and 1-hop regions as the 1-hop connection. The indirect connections between the ROI and 2-hop regions via 1-hop regions are defined as 2-hop connections. According to this logic, when the functional information of all brain regions is unknown, n-hop regions can affect the ROI indirectly and n-hop connections contain functional information of the ROI. We then define the ensemble of 1-hop and 2-hop connections as 2-hops connections. Using these terms, the previous proposal (Passingham et al., 2002) is formulated as that a brain region’s function is represented by the 1-hop connectivity profiles. Separately, based on the above analyses, we proposed that a brain region’s function is represented by the multi-hops connectivity profiles.

To further test our proposal, we selected the right fusiform face area (rFFA) as the ROI, given that this region is the most selective one in the face processing network (Kanwisher et al., 1997). It is advantageous to study individual differences by choosing a region that has a specialized function and is reliably replicated across studies and participants. We adopted the FACES-SHAPES (emotion task) and FACE-AVG (working memory task) contrasts in the human connectome project (HCP) to define individual subject’s functional face activation, and utilized the rfMRI data to construct individual brain functional connectivity network. Inspired by the fact that computations in the graph neural network are analogous to the propagation of functional information in the brain connectivity network, we designed a multi-layer graph neural network (Figure 1B). This graph neural network is well suited for our proposal because it includes both direct and indirect, single-step and multiple-step connectivity features to characterize functional activation of the ROI. Finally, we applied the graph neural network containing the multi-hops functional connectivity to predict individual face activation of the rFFA.

## Materials and Methods

### Human Connectome Project Data

We used the minimally pre-processed data (Glasser et al., 2013) provided by the HCP S1200 release. We selected all the 997 subjects that have the FACES-SHAPES (emotion task) and FACE-AVG (working memory task, AVG represents the average of all other conditions) contrasts, and resting-state fMRI acquisitions.

Task and resting-state fMRI data were projected onto 2 mm standard CIFTI grayordinates space, and the multimodal surface matching (MSM) algorithm (Robinson et al., 2014) based on areal features (MSMAll) was used for accurate inter-subject registration. Acquisition parameters and processing are described in detail in several publications (Barch et al., 2013; Smith et al., 2013). Briefly, resting and task fMRI scans were acquired at 2 mm isotropic resolution, with a fast TR sampling rate at 0.72 s using multiband pulse sequences (Ugurbil et al., 2013). Both sets of functional data had already been registered to the MNI space (Glasser et al., 2013). Each subject had four 15-min resting fMRI runs, with a total of 1,200 time points per run. The resting fMRI data were further pre-processed by ICA-FIX to automatically remove the effect of structured artifacts (Griffanti et al., 2014; Salimi-Khorshidi et al., 2014).

### Functional Connectivity Profile

We calculated functional connectivity based on the HCP-MMP1.0 (Human Connectome Project Multi-Modal Parcellation version 1.0) (Glasser et al., 2016) that contains 360 brain regions. The functional connectivity was calculated from the resting fMRI data. The four runs of individual resting-state time series data were concatenated after being demeaned and variance-normalized along the time axis. We did not apply global signal regression before calculating the functional connectivity. The averaged time series of each brain region was correlated with the averaged time series of the remaining 359 brain regions. The diagonal elements of the functional connectivity matrix were set as ones.

The resulting functional connectivity matrix is dense as there are many small values between brain regions. We did not set any thresholds on the functional connectivity matrix, so as to avoid additional arbitrary choice of parameters. Using a dense network seems to indicate that all brain regions are 1-hop regions and directly connect to the ROI. However, if one also considers the strength factor, many 1-hop connections in the dense network are weak and can be neglected, but the indirect n-hop connections between corresponding regions can be strong. One can understand Figure 1 under the view of connection strength when using the functional connectivity network without thresholds, i.e., each node integrates information from its neighbors with different strengths. Therefore, using a dense functional connectivity network does not harm the definitions of n-hop regions and connections. The definitions of hop in Figure 1 still hold if taking the connection strength into consideration.

### Gaussian-Gamma Mixture Model for Determining Activation Threshold

We calculated each brain region’s activation for each subject by averaging all vertices’ activation within each brain region of HCP-MMP1.0. To prevent some individual differences with opposite signs from canceling with each other, we also calculated each brain region’s mean absolute activation across subjects. Then we used the Gaussian-Gamma mixture distribution (Gorgolewski et al., 2012) to model the density distribution of the mean absolute activation for the 360 brain regions. The density distribution is modeled as a weighted sum of a Gaussian distribution and a Gamma distribution. The Gaussian distribution models the null distribution that represents the noise, and the Gamma distribution models the activation distribution. The mixture model is fitted using an expectation-maximization algorithm. We set the activation threshold as the point where the probability density of Gamma distribution is higher than that of Gaussian distribution.

### Graph Neural Network for Predicting Functional Face Activation

Graph neural network is widely used to process data with graph structures (Defferrard et al., 2016; Kipf and Welling, 2017; Veličković et al., 2018). We developed a graph neural network that is adapted to process brain connectivity network data (Figure 1B). The graph convolution computation in Figure 1C can be realized via a matrix multiplication. The graph neural network with a single-layer can be represented in a matrix form as follows:

(1) $$X_{n\times 1}^{k}=W_{n\times n}^{k}⊙A_{n\times n}X_{n\times 1}^{k-1}+B_{n\times 1}^{k}$$

*k* represents the *k*-th layer of the network. *n* Represents the number of nodes in the graph neural network, i.e., the number of brain regions in the face processing network, and *n* equals 76 or 88 for the face processing network (see details in the section “Functional face activation network selection”). *X* represents the functional activation of each brain region and is a vector with size 76 × 1 or 88 × 1. *B* represents the bias term of the model. *A* represents the adjacency matrix composed of functional connectivity with size 76 × 76 or 88 × 88. *W* has the same size as *A* and represents the functional information propagation coefficient of each functional connectivity pathway. *W* exerts on *A* via the operation ⊙ that represents the element-wise multiplication. The matrix *W* indicates the extent to which each connectivity pathway in *A* involves in the propagation and integration of brain activations. The model realizes the propagation and integration of brain regions’ activations via the matrix multiplication between *W*⊙*A* and *X*. Therefore, the matrix *W* is not symmetrical, with the rows representing integration of information from neighboring regions and the columns representing propagation of information out to neighboring regions. A multi-layer computation can be achieved by applying formula (1) repetitively. The number of parameters in each layer is *n*×*n* (*W*) and *n*×1 (*B*). When one trains a multi-layer graph neural network, the vanishing gradient effect usually occurs. Inspired by the residual neural network (He et al., 2016), we added residual connections between neighboring layers to formula (1) and the resulting model is:

(2) $$X_{n\times 1}^{k}=W_{n\times n}^{k}⊙A_{n\times n}X_{n\times 1}^{k-1}+B_{n\times 1}^{k}+X_{n\times 1}^{k-1}$$

To make the one-layer graph neural network model consistent with the linear model adopted by previous studies, the brain regions’ activation in the 0th layer is initialized as a vector of all ones: $X_{n\times 1}^{0}={\left[1,1,\ldots ,1\right]}_{n\times 1}$. The actual activation is not provided to the model and the initial activation $X_{n\times 1}^{0}$ only serves as a dummy input. The only information we provide to the model is the functional connectivity network. From this point, the previous studies viewed the functional information of neighboring regions identically, but our study estimates the individual differences in neighboring regions via the information propagation in early layers of the multi-layer graph neural network. The rFFA’s functional activation is directly read out from $X_{n\times 1}^{k}$ in the final layer and is used as the final output of the model, with rFFA being one of the n brain regions.

It is worth noting that model (2) does not involve non-linear activation functions but is non-linear in the sense that the functional connectivity network *A* occurs in every computation layer. For instance, the $X_{n\times 1}^{1}=W_{n\times n}^{1}⊙A_{n\times n}X_{n\times 1}^{0}$ contains linear features in *A*, but the $X_{n\times 1}^{2}=W_{n\times n}^{2}⊙A_{n\times n}X_{n\times 1}^{1}$ involves the matrix multiplication between *A* and *X*^1^ and contains second-order features in *A*. Therefore, the resulting multi-layer model contains non-linear features in *A* that represent functional information passing through multiple-step functional connectivity pathways.

### Multi-Hop Connections Represented in the Graph Neural Network

In Figure 1A, direct neighbors of the ROI are defined as 1-hop regions and the direct neighbors of 1-hop regions are defined as 2-hop regions relative to the ROI; 1-hops regions directly connect to the ROI and can affect the ROI via 1-hop connections, and 2-hop regions do not directly connect to the ROI but can affect 1-hop regions via connections between 1-hop and 2-hop regions. Therefore, 2-hop regions can indirectly affect the ROI via 1-hop regions and these indirect effects are defined to transfer via 2-hop connections. In the multi-layer graph neural network model (Figure 1B), indirect effects transferred via 2-hop connections are represented by the multiplication between 1-hop connections and the connections between 1-hop and 2-hop regions. In the graph convolution computation (Figure 1C) within each layer, each region integrates functional information from 1-hop regions. But in the consecutive propagation of information in an n-layer graph neural network, the information of n-hop regions can transfer to the ROI via n-hop connections. Even though the model is still linear with respect to the initial activation, the initial activation is only a dummy input that propagates within the brain network to predict the actual brain activation. The model parameters determine how the initial activation propagates within the brain network and the important feature of the model is the functional connectivity network. Whether the model is nonlinear or not should be determined on the functional connectivity rather than the dummy initial activation.

### Metrics for Assessing the Model

We used two metrics to assess the individual prediction performance on testing data. Denote the target value by *y* and the prediction value by $\hat{y}$. The sum squared error (SSE) = $\sum_{i}{\left(y_{i}-{\hat{y}}_{i}\right)}^{2}$ is widely used to assess the difference between the target and prediction values (index *i* runs over all testing subjects). But in different divisions of the dataset, the variance of the target value is different, thus the SSE cannot be compared across different divisions. We divided the SSE by the sum squared total (SST) = $\sum_{i}{\left(y_{i}-\text{mean}\left(y\right)\right)}^{2}$ and the resulted normalized squared error (NSE) was used. The NSE value is 0 when the model achieves perfect individual prediction, and larger NSE values indicate lower performance. We also adopted the Pearson correlation to assess the similarity between each testing subject’s actual and prediction value, denoted by *r*. Because the correlation is calculated across subjects, the value of *r* is 1 when the model achieves perfect individual prediction, and the value of *r* is 0 or negative when the model achieves poor individual prediction. Under the least squares condition, NSE represents the error proportion that cannot be explained by the model, and *r*^2^ represents the proportion of target data that can be explained by the model. Since the least squares condition is not satisfied by our model, these two metrics only serve as approximations.

Prediction similarity assessed by the Pearson correlation coefficient was Fisher’s *z* transformed when used for further statistical tests. Since the evaluation metrics for different models were paired for each random division of the dataset, we performed paired-sample *t*-tests using the custom Matlab command “ttest.”

### Implementation Details

The whole dataset was randomly divided into a training set and a testing set with a ratio of nine to one. Though the sample size 997 is relatively large in neuroimaging, it is rather small compared to that of computer vision datasets in machine learning that usually contain more than 10,000 samples. The random splitting of a small dataset can introduce random effects into the final results, i.e., the metrics for assessing the model can vary widely across different divisions. To remove the random effect as much as possible, we performed the prediction process 100 times with different random divisions of the dataset and used the mean of the two metrics to assess the model.

We implemented the graph neural network with PyTorch^1^. The parameters of the model were initialized by the Xavier normal distribution with a gain of 0.1. The model was trained via the stochastic gradient descent optimizer to minimize the NSE with a Nesterov momentum of 0.9 used. The training batch size was 128 and 500 training epochs were used. The initial learning rate was 0.01 and a 0.1 multiplicative factor of learning rate decay was set at 300 and 400 epochs respectively. To further overcome the problem of overfitting caused by a small sample size, we added Gaussian random noise to the individual connectivity features at each training step. The variance of the Gaussian random noise was set equal to the variance of each connectivity feature across subjects. This technique can be viewed as a kind of online data augmentation. The graph neural network models were trained on an NVIDIA GeForce GTX 1080 Ti graphic processing unit. The training time for each random separation lasts about 8 min, and the total training time for 100 random separations lasts about 13 h.

## Results

### Functional Face ROI Localization

**FIGURE 2:**
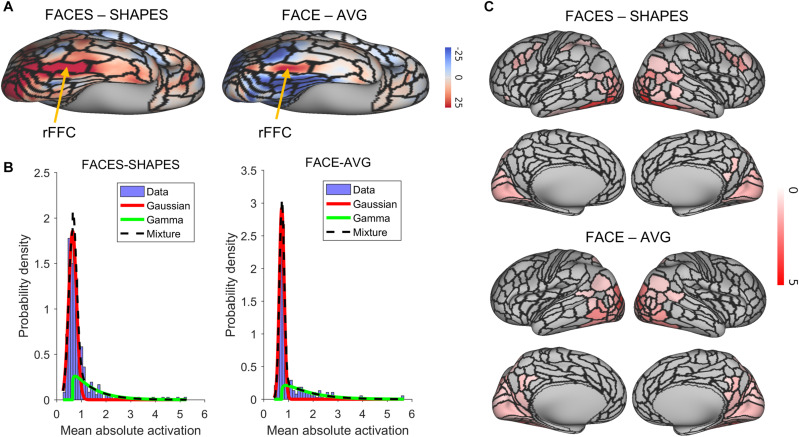
Functional face ROI localization and network selection. **(A)** Voxel-wise group average *z*-statistics of both contrasts were shown. The yellow arrow indicates the area where the rFFC region locates. **(B)** The purple histogram indicates the density distribution of the mean absolute activation for the 360 brain regions. A Gaussian (red curve)-Gamma (green curve) mixture (black curve) model was used to fit the data. **(C)** Mean absolute *z*-statistics of brain regions in the functional face activation network were shown.

We identified the rFFA by the right fusiform face complex (rFFC) region in the HCP-MMP1.0 (Glasser et al., 2016). The FACES-SHAPES task contrast for the HCP emotion paradigm and the FACE-AVG task contrast for the HCP working memory paradigm were separately used to identify the functional face activation. We showed the group average *z*-statistics of these two task contrasts and the boundary of rFFC region in Figure 2A. The rFFC region was identified in both task contrasts, and the boundary of rFFC region coincided well with that of the group average activation. The mean *z*-statistic within the rFFC region was used to assess each subject’s face activation.

### Functional Face Activation Network Selection

We next defined the functional face activation network in preparation for constructing the graph neural network. The HCP_MMP1.0 contains 360 brain regions. Using a connectivity matrix with size 360 × 360 is likely to overfit the training dataset. Since some brain regions do not involve in the face recognition process, removing these brain regions beforehand can reduce the model complexity in a great deal. Figure 2B shows the density distribution of the mean absolute activation for the 360 brain regions. We used a Gaussian-Gamma mixture model (described in the section “Materials and Methods”) to select the activation networks. The selection of task-related regions is independent of the end-to-end training procedure of the graph neural network, since the dummy input of the network is initialized as a vector of ones and cannot be used for selecting the activation network. The number of remaining brain regions for the FACES-SHAPES contrast is 76, and that for the FACE-AVG contrast is 88, resulting in connectivity matrices with size 76×76 and 88×88 respectively. We showed the activation networks of both task contrasts in Figure 2C. Networks of both task contrasts mainly include brain regions in the visual cortices, such as the primary and early visual cortices, dorsal and ventral stream visual cortices, MT + complex and neighboring visual areas. Both networks also include medial and lateral temporal cortices, superior and inferior parietal cortices, temporo-parieto-occipital junction, and posterior cingulate. In addition, the activation network of FACES-SHAPES contrast also includes inferior frontal, orbital and polar frontal, dorsolateral prefrontal, and premotor cortices. The activation network of FACES-SHAPES contrast is broader than that of FACE-AVG contrast, because the activation network of FACE-AVG contrast is mainly for basic face perception, while the activation network of FACES-SHAPES contrast also includes emotional processing of faces.

### Statistical Validation of Graph Neural Network Prediction Model

**FIGURE 3:**
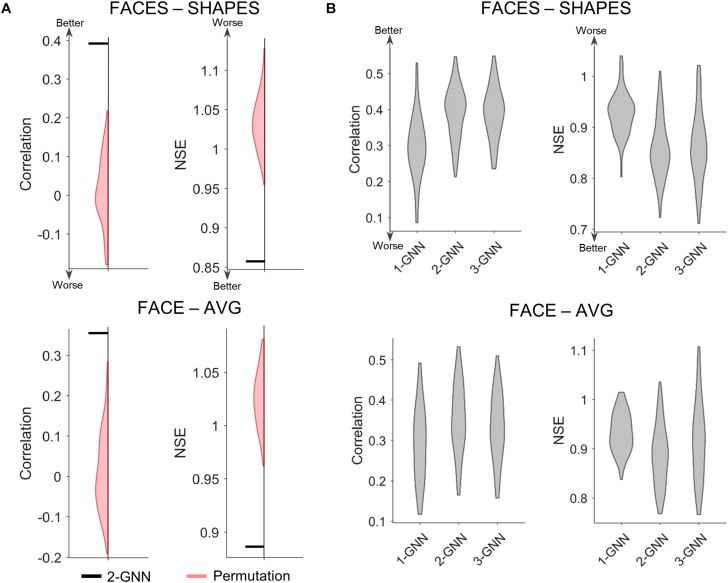
Comparison of prediction metrics for different models. **(A)** Comparison between the two-layer graph neural network (2-GNN) and the random permutation model with the same structure. The prediction performance of 2-GNN is better than that of random permutation model in that the mean NSE of 2-GNN is below the distribution of permutation and the mean correlation of 2-GNN is above the distribution of permutation. **(B)** Comparison of graph neural networks with different layers. The 2-GNN has a higher ability to predict better individual differences than both the 1-GNN and 3-GNN.

After selecting the functional face activation network, we constructed graph neural networks to predict individual face activation of the rFFC region. We first compared the two-layer graph neural network with the random permutation model to validate the prediction model statistically. Since we intended to test whether the individual association between functional connectivity network and the face activation of rFFC region is significant, the random permutation model was re-trained with the same structure as the two-layer graph neural network, except that the pairings between the functional connectivity network and the activation of rFFC region were shuffled. We did 1,000 random permutations and calculated whether the mean prediction accuracy of the two-layer graph neural network model was better than the 99th percentile of the prediction accuracy of the random models. We illustrated the comparison between the two-layer graph neural network and the random permutation model in Figure 3A. For both the FACES-SHAPES and FACE-AVG contrast, the mean NSE of two-layer graph neural network is far below the distribution of random permutation model, and the mean correlation of two-layer graph neural network is far above the distribution of random permutation model. Hence, the two-layer graph neural network can capture the association between the individual functional connectivity network and the face activation of rFFC region above random level.

### Comparison of Graph Neural Networks With Different Layers

After validating the graph neural network statistically, we further tested our proposed assumption by comparing graph neural networks with different layers. The one-layer graph neural network corresponds to the linear prediction model adopted by previous studies and utilizes the 1-hop (i.e., direct) functional connectivity of the rFFC region to predict the rFFC region’s individual functional face activation. In the two-layer graph neural network, the final layer corresponds to the 1-hop functional connectivity representation of the rFFC region’s face activation, and the first layer corresponds to the 2-hop functional connectivity representation of the rFFC region’s face activation. Thus, the multi-layer graph neural network contains the representation of the rFFC region’s face activation through multi-hops functional connectivity. If the proposal that a brain region’s function is represented by the multi-hops connectivity is rational, using multi-hops functional connectivity should improve the prediction of the rFFC region’s face activation. We determined the rational number of hops based on the generalization ability of the graph neural networks with different numbers of layers and showed the comparison results in Figure 3B and Supplementary Table S1. For the FACES-SHAPES contrast, the NSE of two-layer graph neural network (mean NSE = 0.857) is significantly [*t*(99) = −16.0, *p* = 3.0 × 10^–29^, paired-sample *t*-test] lower than that of one-layer graph neural network (mean NSE = 0.927), but is not very significantly [*t*(99) = −2.09, *p* = 0.039, paired-sample *t*-test] lower than that of three-layer graph neural network (mean NSE = 0.864). The correlation of two-layer graph neural network (mean correlation = 0.392) is significantly [*t*(99) = 13.5, *p* = 2.8 × 10^–24^, paired-sample *t*-test] higher than that of one-layer graph neural network (mean correlation = 0.296), but is not significantly [*t*(99) = 0.269, *p* = 0.788, paired-sample *t*-test] higher than that of three-layer graph neural network (mean correlation = 0.391). Results for the FACE-AVG contrast are similar, except that the differences between the two-layer and three-layer graph neural networks are significant (see Supplementary Table S1). Since the evaluation metrics stopped improving and adding more layers leads to worse generalization performance, we only tested graph neural networks with the number of layers up to 3. Overall, the multi-layer graph neural network improves the prediction performance in individual face activation of the rFFC region, and the two-layer graph neural network possessed the best prediction performance.

### Functional Network Pathways Involving rFFC’s Face Function

**FIGURE 4:**
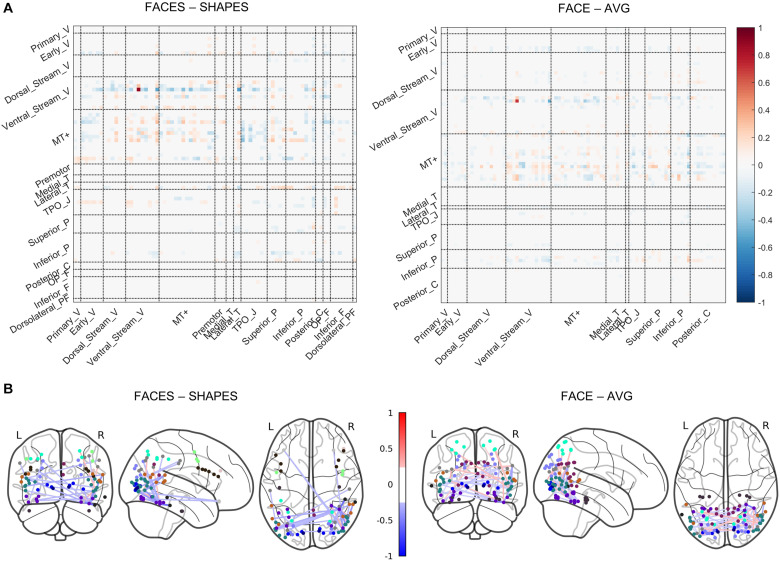
Visualization of the propagation coefficient involving rFFC’s face function. The propagation coefficient *W* in the first layer of the network is shown. **(A)** The coefficients are shown in a matrix plot. Color represents the coefficient strength of each connection. Brain regions that belong to the same cortex are grouped. Full names of cortices are included in Supplementary Table S2. **(B)** The coefficients are plotted on the glass brain. Only the connection with the top 1% coefficient strength is shown to make the plot clean. Edge color represents the coefficient strength of each connection. Node color represents the cortices that each brain region belongs to (see Supplementary Figure S1).

In the previous section, we determined that the two-layer graph neural network has the best generalization ability, thus the functional network pathways containing the rFFC region’s 1-hop and 2-hop functional connectivity best characterize the individual functional face activation of the rFFC region. We utilized the functional information propagation coefficient *W* in the first layer to analyze the functional network pathways involving face processing (Figure 4). The propagation coefficient *W* in the first layer also contains information about the *W* in the second layer, because the output of the first layer is subsequently used as the input of the second layer. The propagation coefficient *W* is not symmetrical. The rows represent brain regions that integrate functional information from neighboring regions, and the columns represent brain regions that send functional information out. The coefficients are different for the two task contrasts because the two tasks have different functional activation networks. Nonetheless, in both task contrasts, coefficients with large absolute values mainly concentrate in the rows of ventral stream visual cortices and MT + complex visual areas. This result indicates that brain regions in these two cortices mainly participate in the computation of the following layer. Though some brain regions in other rows do not have large absolute connectivity coefficients, these regions in the columns have large absolute connectivity coefficients. This result indicates that some brain regions do not integrate functional information for the following layer, but they send functional information out to the regions that integrate functional information. The whole results suggest a hierarchical functional face processing mechanism for the rFFC region. The rFFC region first mainly integrates functional information from regions in the ventral stream visual cortices and MT + complex visual areas, then regions in these two cortices integrate functional information from regions in other cortices.

## Discussion

In order to better characterize the brain region’s function of individuals, our study proposed that a brain region’s function is represented by the multi-hops connectivity profiles. The multi-layer graph neural network model was used to incorporate multi-hops connectivity features in the functional connectivity network. We tested our proposal by predicting the functional face activation of the rFFC region via the rFFC region’s multi-hops functional connectivity. Our results showed that the 2-hops functional connectivity profile has the best generalization ability in characterizing the rFFC region’s individual functional face activation, and revealed a hierarchical network for the rFFC region’s functional face processing mechanism. The current study provides new insights into understanding the brain region’s function from a network perspective.

Previous researchers proposed that a brain region’s function is represented by the 1-hop connectivity profiles (Passingham et al., 2002). However, this proposal neglects individual differences in the functional information of ROIs’ neighboring regions. Under our proposal that a brain region’s function is represented by the multi-hops connectivity profiles, individual differences in the 1-hop brain region’s functional information are taken into consideration via the 2-hop connectivity profiles. Our proposal is also consistent with neuroscience findings. Researchers have suggested that brain functions do not rely on the independent operation of a single brain region or connectivity pathway, but derive from the brain network composed of multiple brain regions and connectivity pathways (McIntosh, 2000; Misic and Sporns, 2016). In addition, indirect connectivity features among other brain regions can also affect ROIs via the brain network (Honey et al., 2009). Brain navigation efficiency is also due to multi-hop brain connectivity pathways (Seguin et al., 2018). In a word, since the multi-hop connectivity encodes the topological and geometrical properties of the brain connectivity network, our proposal indicates that a brain region’s function is encoded in the topology of the brain connectivity network.

The multi-layer graph neural network perfectly matches our proposal, since the multi-layer convolution computations characterize the propagation of functional information among the brain connectivity network. Though some kinds of graph neural network models have been developed to process brain network data (Ktena et al., 2018; Parisot et al., 2018; Yang et al., 2019; Kim and Ye, 2020), our proposed graph neural network is novel in the following aspect. As opposed to these graph neural networks (Ktena et al., 2018; Parisot et al., 2018; Yang et al., 2019; Kim and Ye, 2020) that either impose feature transformation parameters on node features or use graph attentions that utilize node features to construct network propagation coefficients, our graph neural network directly imposes parameters on the connectivity network matrix instead. Imposing parameters on the connectivity network matrix is especially beneficial when the dimension of node features is very low, as it is the case that the node feature, i.e., the brain activation statistic, has only one dimension in our study. Hence, our graph neural network is well suited for handling connectivity-driven problems, while the others mainly aim at dealing with node-driven problems.

The functional connectivity network has also been verified to transfer functional information across cortical regions (Cole et al., 2016; Ito et al., 2017). Under this activity flow mapping, functional activation information is transferred to neighboring brain regions via functional connectivity pathways. The activity flow mapping shares certain similarities with our study in the sense that the functional information propagates within the functional connectivity network. However, our study differs from the activity flow framework mainly in that functional activation information of all brain regions in our study is unknown, while only the ROI’s functional activation information is unknown in the activity flow framework. In this sense, our proposal and study require less functional information of brain regions and thus has practical implications in that one does not need to scan functional task contrasts of unseen subjects beforehand to get the functional information of some brain regions after training the model that utilizes the same task.

Our results showed that the two-layer graph neural network containing 1-hop and 2-hop functional connectivity best characterizes the rFFC region’s functional face activation, indicating that 2-hops connectivity information may be enough to estimate the rFFC region’s function. On the other hand, from the computation perspective, as the number of layers in a graph neural network gets large, the parameters and complexity of the model also enlarge. Since the sample size is relatively limited compared to that of datasets in machine learning, models with large complexity are also likely to overfit the data and thus have a poor generalization ability. Future work involves utilizing datasets with a large sample size to test whether graph neural networks with more layers can further improve the generalization ability.

We chose the rFFC region that has a specialized function and is reliably replicated across studies to test our assumption primarily. However, the rFFC region is specialized in the face function, which has special meaning in the human evolution process and has a specific neural mechanism (Tsao et al., 2006; Freiwald and Tsao, 2014). Whether our proposal can be generalized to brain regions beyond the rFFC still remains to be solved, especially to brain regions that are more functionally variable across individuals and flexible across tasks, i.e., the heteromodal association cortices (Anderson et al., 2013; Mueller et al., 2013; Tei et al., 2017). Future work also includes extension to brain regions involving wide functional domains to test our proposal.

We used undirected functional connectivity to construct the brain connectivity network in this study. However, the propagation of functional information in the brain is actually directional, and this directional information was not taken into account. Effective connectivity should be considered in the future to capture the directionality of information transfer. In addition, there are also other choices to construct the brain connectivity network, such as the structural connectivity representing white matter fiber pathways. Researchers can also explore the relationship between the multi-hops structural connectivity network and the individual brain region’s function.

## Conclusion

We proposed that the multi-hops connectivity profile can improve the prediction performance of individual differences in the brain region’s function. Results revealed that the 2-hops functional connectivity network best characterizes the rFFC region’s individual functional face activation. This advancement contributes to understanding the mechanism of individual brain region’s function in terms of the brain network and provides a new perspective on brain functional processing mechanisms at the network level.

## Data Availability Statement

The HCP S1200 data release is publicly available online at https://www.humanconnectome.org/. The code for performing the graph neural network is available from https://github.com/allizwell2018/Multi-hops-connectivity.

## Ethics Statement

The studies involving human participants were reviewed and approved by NIH Neuroscience Blueprint Institutes and Centers. The patients/participants provided their written informed consent to participate in this study.

## Author Contributions

DW, XL, and JF designed the research, wrote and revised the manuscript. DW performed the data analysis. All the authors contributed to the article and approved the submitted version.

## Conflict of Interest

The authors declare that the research was conducted in the absence of any commercial or financial relationships that could be construed as a potential conflict of interest.

## Footnotes

1 https://pytorch.org/
