# Supplementary material for: Multi-Hops Functional Connectivity Improves Individual Prediction of Fusiform Face Activation via a Graph Neural Network
Source: Front Neurosci. 2021 Jan 14;14:596109. doi: 10.3389/fnins.2020.596109 (PMC7840579; doi:10.3389/fnins.2020.596109)
Supplement: Supplementary file 1 [file Data_Sheet_1.pdf]

Supplementary Material

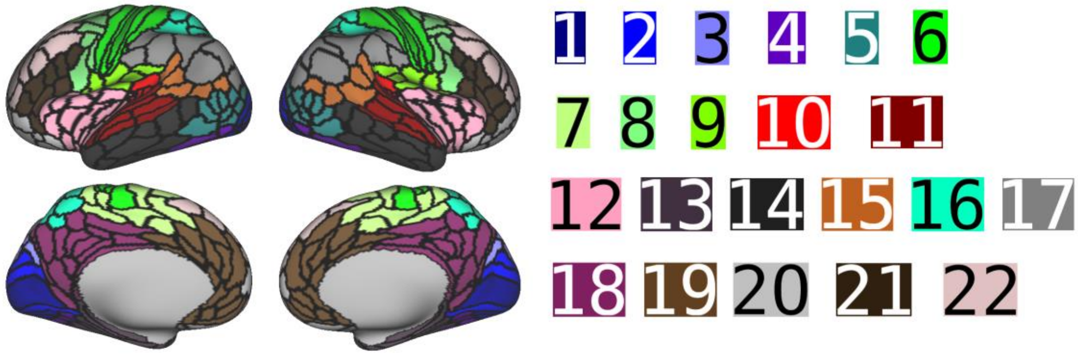

Figure S1. Color coding of the brain regions. The cortical names corresponding to the numbers are shown in Table S2. This figure is adapted from Figure 1 in the Supplementary Neuroanatomical Results for A Multi-modal Parcellation of Human Cerebral Cortex.

Table S1. Statistical comparison of the prediction metrics. The mean prediction metrics and the statistical comparison of metrics between different models were shown. The k-GNN represents the graph neural network with k layers. NSE is the abbreviation of normalized squared error.

**FACES-SHAPES**		
**Mean**	NSE	Correlation
1-GNN	0.927	0.296
2-GNN	0.857	0.392
3-GNN	0.864	0.391
**Paired-sample *t*-test**	NSE	Correlation
2-GNN/1-GNN	$t(99) = -16.0$, $p = 3.0 \times 10^{-29}$	$t(99) = 13.5$, $p = 2.8 \times 10^{-24}$
2-GNN/3-GNN	$t(99) = -2.09$, $p = 0.039$	$t(99) = 0.269$, $p = 0.788$
3-GNN/1-GNN	$t(99) = -10.4$, $p = 1.6 \times 10^{-17}$	$t(99) = 11.5$, $p = 4.7 \times 10^{-20}$
**FACE-AVG**		
**Mean**	NSE	Correlation
1-GNN	0.933	0.291
2-GNN	0.886	0.355
3-GNN	0.915	0.332
**Paired-sample *t*-test**	NSE	Correlation
2-GNN/1-GNN	$t(99) = -11.3$, $p = 1.4 \times 10^{-19}$	$t(99) = 9.29$, $p = 3.8 \times 10^{-15}$
2-GNN/3-GNN	$t(99) = -7.93$, $p = 3.4 \times 10^{-12}$	$t(99) = 5.96$, $p = 3.9 \times 10^{-8}$
3-GNN/1-GNN	$t(99) = -2.70$, $p = 0.008$	$t(99) = 4.65$, $p = 1.0 \times 10^{-5}$

Table S2. Full names of the abbreviations used in Figure 4. Only the cortices that appeared in the article are abbreviated. These names were used in the Supplementary Neuroanatomical Results for A Multi-modal Parcellation of Human Cerebral Cortex.

Number of cortex	Full name	Abbreviation
1	Primary_Visual	Primary_V
2	Early_Visual	Early_V
3	Dorsal_Stream_Visual	Dorsal_Stream_V
4	Ventral_Stream_Visual	Ventral_Stream_V
5	MT+_Complex_and_Neighboring_Visual_Areas	MT+
6	Somatosensory_and_Motor	-
7	Paracentral_Lobular_and_Mid_Cingulate	-
8	Premotor	Premotor
9	Posterior_Opercular	-
10	Early_Auditory	-
11	Auditory_Association	-
12	Insular_and_Frontal_Opercular	-
13	Medial_Temporal	Medial_T
14	Lateral_Temporal	Lateral_T
15	Temporo-Parieto-Occipital_Junction	TPO_J
16	Superior_Parietal	Superior_P
17	Inferior_Parietal	Inferior_P
18	Posterior_Cingulate	Posterior_C
19	Anterior_Cingulate_and_Medial_Prefrontal	-
20	Orbital_and_Polar_Frontal	OP_F
21	Inferior_Frontal	Inferior_F
22	Dorsolateral_Prefrontal	Dorsolateral_PF
